# Association of Life's Essential 8 with osteoarthritis in United States adults: mediating effects of dietary intake of live microbes

**DOI:** 10.3389/fmed.2023.1297482

**Published:** 2023-12-21

**Authors:** Ruoyu Gou, Xiaoyu Chang, Zeyuan Li, Ying Pan, Guanghua Li

**Affiliations:** ^1^School of Public Health, Ningxia Medical University, Yinchuan, Ningxia, China; ^2^Department of Joint Surgery, HongHui Hospital, Xi'an Jiaotong University, Xi'an, Shannxi Province, China

**Keywords:** osteoarthritis, Life's Essential 8, mediation analyses, live microbes, NHANES

## Abstract

**Objective:**

Osteoarthritis (OA) is associated with cardiovascular disease and represents a persistent economic and physical burden on patients in the United States. This study evaluated the mediating effect of dietary live microbe intake on the association between cardiovascular health [based on Life's Essential 8 (LE8) scores] and osteoarthritis (OA) in adults.

**Methods:**

This cross-sectional study included data from the National Health and Nutrition Examination Survey, 1999–2019 (from patients aged ≥20 years). LE8 scores (0–100) were measured according to the American Heart Association definition and categorized as low (0–49), moderate (50–79), or high (80–100). OA disease status was assessed using self-reported data from patients. The relationships were evaluated using multivariate logistic and restricted cubic spline models. Mediation analysis was used to evaluate the mediating effect of dietary live microbe intake on the association between LE8 and OA risk.

**Results:**

The study included 23,213 participants aged ≥20 years. After adjusting for latent confounders, higher LE8 scores were found to be associated with a lower incidence of OA. The odds ratios (with 95% confidence intervals) for low, moderate, and high OA risk were 0.81 (0.69, 0.96) and 0.55 (0.44, 0.69), respectively; a non-linear dose-response relationship was observed (*P*-nonlinear = 0.012). Health behavior and health factor scores showed a similar pattern of correlation with OA risk. Low live microbe intake mediated the association between LE8, health behavior, and health factor scores with OA risk and did not appear to reduce OA risk.

**Conclusion:**

Our findings suggest that although higher LE8 scores reduce the risk of developing OA, low live microbe intake may reduce the protective effect of higher scores. It is, therefore, essential to emphasize adherence to a lifestyle that confers high LE8 scores. Individuals should also be advised to reduce the intake of foods with low live microbe content.

## 1 Introduction

Osteoarthritis (OA) is the most common form of arthritis and is caused by an imbalance between repair and destruction of joint tissue. It entails structural changes in the joints, including cartilage degeneration, synovial inflammation, and inflammation of the capsular ligaments. The United States has the highest age-standardized prevalence rate of OA (1), and the number of affected individuals is expected to increase to 67 million by 2030 (2). Notably, OA is associated with an increased risk of premature death from cardiovascular disease (CVD). Therefore, healthcare professionals need to take particular note of modifiable cardiovascular risk factors (including hypertension, diabetes mellitus, hyperlipidemia, smoking, and physical inactivity) in this population (3). Certain lifestyle-related risk factors have been demonstrated to have a definite biological effect on the development of OA and these include age, gender, smoking, diet, hypertension, sedentary lifestyle, body mass index (BMI), low-density lipoprotein levels, genetics, metformin use, bone mineral density, joint shape abnormalities, joint malalignment, decreased muscle strength/mass, injuries, and joint loading abnormalities (4–6). Although the causes of OA have not been fully elucidated, there is a growing agreement that indispensable environmental factors (health behaviors and diet) are important contributors to this disease (6, 7). In 2010, the American Heart Association recommended Life's Simple 7 as a measure of cardiovascular health (CVH), with the aim of improving the health of the general population (8). Owing to the limitations of the LS7 CVH score, the American Heart Association recently updated the evaluation tool to Life's Essential 8 (LE8) (9). The LE8 scoring system is more sensitive to differences between individuals and emphasizes the role of maintaining or improving CVH. The components of LE8 include diet (updated), physical activity, nicotine exposure (updated), sleep health (new), body mass index, blood lipids (updated), blood glucose (updated), and blood pressure. In this context, epidemiologic studies have shown that conventional risk factors for CVD such as age, hypertension, diabetes mellitus, obesity, and low physical activity are associated with the development and progression of OA (10). However, no studies have evaluated the association between CVH (LE8 scores) and OA risk.

Notably, previous studies have shown the existence of mutual protective factors between OA and CVD (11). In their study, Sanders et al. assessed the number of live microbes consumed in the diet and accordingly categorized foods into low [Lo; < 104 colony forming units (CFU)/g], medium (Med; 104–107 CFU/g), and high (Hi: >107 CFU/g) groups based on the number of live microbes per gram. In this context, certain probiotics (including *Bifidobacterium bifidum* and *Lactobacillus acidophilus*) may lower elevated cholesterol levels and aid the prevention and treatment of some CVDs (12, 13). Beneficial symbiotic microbes have also been found to be capable of exerting cholesterol-lowering effects (14). In addition, studies have reported that gut dysbiosis exacerbates OA; this may be explained by disruption of the host–gut microbial balance, which, in turn, triggers the host immune response and activates the “gut–joint axis” (15). Research suggests that LE8 scores and live microbe intake may reduce the risk of OA by reducing oxidative stress, inflammation, and obesity (16). However, the relevance of these factors is limited by the lack of animal and human studies on this association. This cross-sectional study, using data from the National Health and Nutrition Examination Survey (NHANES) 2005–2019 cohort, was therefore performed to evaluate the association between LE8 scores and OA risk. The live microbe content of 9,388 foods listed in the NHANES database was also evaluated; the foods were grouped according to microbial content, and their mediating effects were assessed.

## 2 Materials and methods

### 2.1 Database and study subjects

**Figure 1:**
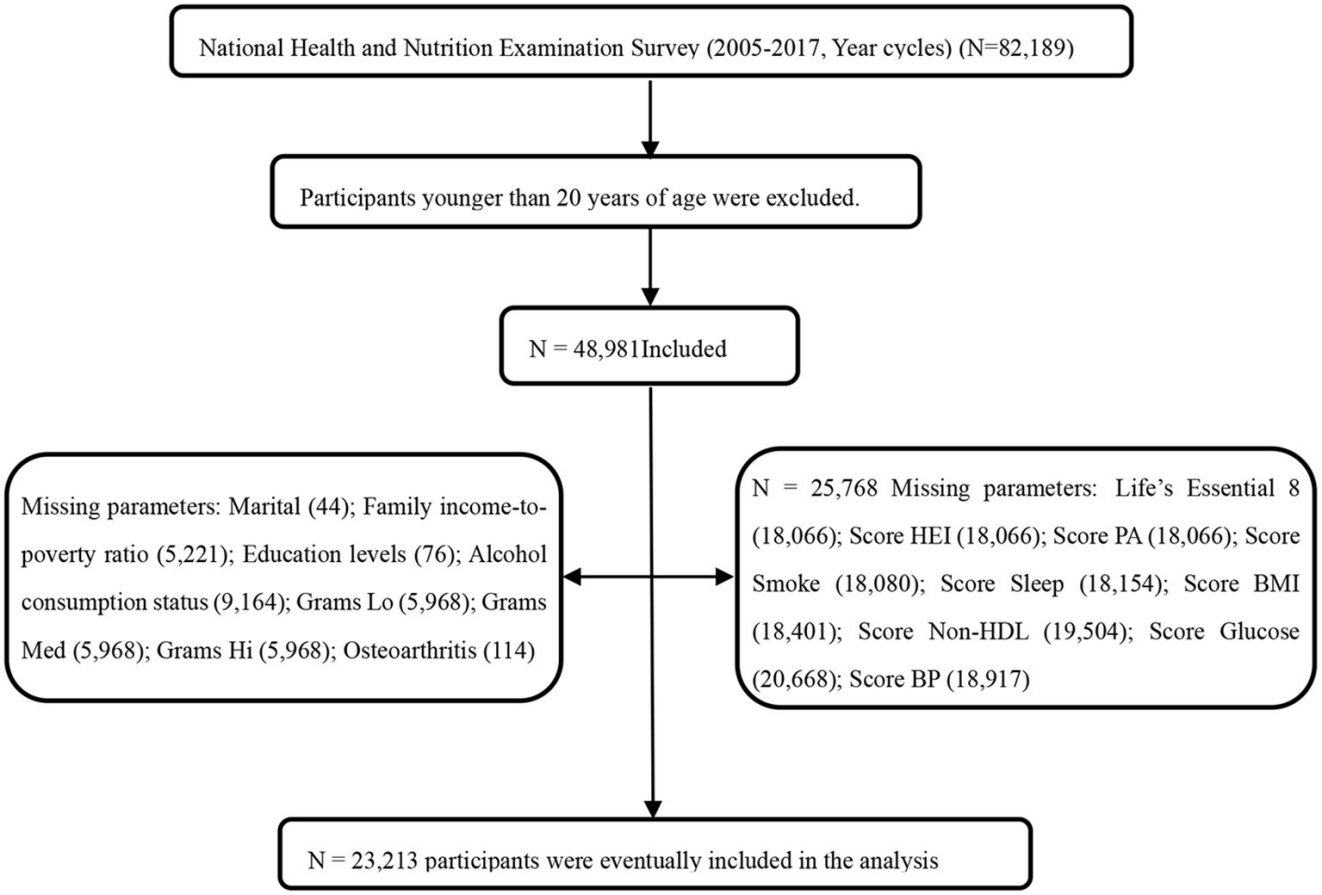
Flowchart of the screening process for the selection of the study population. NHANES, National Health and Nutrition Examination Survey; FBG, fasting blood glucose; HEI score, Healthy Eating Index; Non-HDL, Non-High-Density Lipoprotein Cholesterol; BP, Blood Pressure; Experts assigned foods an estimated level of live microbes per gram [low (Lo), < 104 CFU/g; medium (Med), 104–107 CFU/g; or high (Hi), >107 CFU/g]; CVH, cardiovascular health; Life's Essential 8, The components of Life's Essential 8 include diet (updated), physical activity, nicotine exposure (updated), sleep health (new), body mass index, blood lipids (updated), blood glucose (updated), and blood pressure. Each metric has a new scoring algorithm ranging from 0 to 100 points, allowing the generation of a new composite cardiovascular health score (the unweighted average of all components) that also varies from 0 to 100 points. The LE8 scoring algorithm consists of 4 health behaviors (diet, physical activity, nicotine exposure, and sleep duration) and 4 health factors [body mass index (BMI), non-high-density-lipoprotein cholesterol, blood glucose, and blood pressure]. The overall LE8 score, health behavior score, and health factor score were calculated as the unweighted average of the eight metrics. Participants with a LE8 score of 80–100 were considered high CVH; 50–79, moderate CVH; and 0–49 points, low CVH.

The NHANES utilizes stratified multistage probability sampling methods to select a series of nationally representative samples of non-institutionalized United States adults in 2-year cycles, which started from 1999 to 2000 (http://www.cdc.go/nchs/nhanes.htm). The NHANES program was approved by the Ethics Review Board of the National Center for Health Statistics. All participants provided written informed consent to participate in the survey and agreed to the use of their data in health-related statistical research. The study followed the Strengthening Reporting of Observational Studies in Epidemiology reporting guidelines (17). As shown in Figure 1, data from 23,213 participants (aged 20 years and older with complete data from eight survey cycles spanning from 2005–2006 to 2017–2019) were included in the study.

### 2.2 Measurement of LE8 scores

The LE8-scored sub-questionnaire assesses four health behaviors (diet, physical activity, nicotine exposure, and sleep duration) and four health factors (BMI, non-high-density lipoprotein cholesterol, blood glucose, and blood pressure) (9). The scores for the eight CVH metrics range from 0 to 100 (9). The overall LE8 score is calculated as the arithmetic mean of the eight metrics. Participants with LE8 scores of 80–100, 50–79, and 0–49 are considered to have high, moderate, and low CVH, respectively. In this study, dietary indicators were evaluated using the Healthy Eating Index 2015 scores; dietary intakes of participants (obtained from two 24-h dietary recalls) were combined with United States Department of Agriculture Food Pattern Equivalent data to calculate the scores. Self-reported questionnaires were used to obtain information regarding the frequency and duration of vigorous or moderate physical activity over the past 30 days. Smoking habits, sleep duration, history of diabetes, and medication history were also assessed using self-reported questionnaires. Blood pressure, height, and weight were measured during physical examination. The blood pressure was determined by averaging three consecutive measurements, and the BMI was calculated by dividing the weight in kilograms by the square of the height in meters. Blood samples were collected and sent to a central laboratory for analysis of lipid, blood glucose, and glycosylated hemoglobin levels (9).

### 2.3 OA assessment

A study has shown 81% consistency between self-reported and clinically confirmed diagnoses (18). In the NHANES, OA was diagnosed by a professional, and information was obtained using a questionnaire. All participants (aged ≥20 years) were asked questions related to arthritis. They were asked the following question: “Has a doctor or other health professional ever told you that you have arthritis?” Participants were included in the study if their responses indicated that they were diagnosed with OA (19).

### 2.4 Definition of live microbes in food

A classification system has been established for defining and estimating the dietary intake of live microbes among United States adults. The NHANES uses food codes for the assigned categories. Among the 9,388 food codes in the NHANES database, 8,954 contain small amounts of live microbes (< 104 CFU/g) (20). Processed foods that usually undergo heat treatment (such as milk; prepared meats including pork, poultry, and seafood dishes; and sauces and gravies) are considered to be very low in microbes and are therefore classified as Lo foods. Raw meats, pork, poultry, and seafood are also classified in the Lo category, based on the presumption that they are cooked prior to consumption (with the exception of a few of these foods that are specified as being eaten raw). Fresh vegetables and fruits are the top two food items allocated to the Med category (accounting for 41 and 39%, respectively). Fresh fruit juices, such as fruit smoothies, are allocated to the Med category; beverages, condiments, and sauces comprise more than 10% of the food allocated to this category. Some fermented foods (e.g., miso and sauerkraut) are also assigned to the Med category. Notably, fermented dairy products comprise the majority of foods assigned to the Hi category. Yogurt and other fermented milks are assigned to this category unless they constitute a component of another food. Codes that contain a significant amount of fermented foods, such as yogurt or sour cream, are assigned to the Hi category. However, foods that contain cheese as a minor component are assigned to the Lo or Med categories, depending on their relative amount in the food (20).

### 2.5 Defining covariates

Demographic information was obtained using a questionnaire. The attributes included age (20–44, 45–64, and ≥65 years), gender, race (non-Hispanic white, non-Hispanic Black, Hispanic, and others), marital status (married, separated, and never married), and family income to poverty ratio (< 1.30, 1.30– < 3.00, 3.00– < 5.00, and ≥5.00). This ratio represents the proportion of the family income in relation to the federal poverty threshold after adjusting for household size; a higher ratio indicates a higher level of income. Data were also obtained on the level of education [less than high school (grade 11), high school graduate/general education, college, and above college level] and the alcohol consumption status [current heavy drinker (women: ≥three drinks per day, men: ≥four drinks per day, or binge drinking for 5 days or more per month), current moderate drinker (women: ≥two drinks per day, men: ≥three drinks per day, or binge drinking ≥2 days per month), current light/mild alcohol drinkers (not fulfilling the criteria for the previous two categories) (6), former alcohol consumption (previous history of drinking but not a current drinker), and no history of alcohol consumption].

### 2.6 Statistical analysis

To estimate the statistical data representative of United States adults, oversampling, stratification, and clustering were performed in accordance with the NHANES guidelines; particular emphasis was placed on weight-adjusted statistical tests. Chi-square and *t*-tests were used to evaluate demographic characteristics, pertaining to OA status. The association between LE8 scores and the risk of OA was evaluated using a multivariate logistic regression model; the results were presented as the odds ratio (OR) with 95% confidence intervals (95% CIs). Stratified analyses were performed by gender, age, and ethnicity to evaluate the association between LE8 scores and OA risk in different groups. The group with low LE8, health behavior, and health factor scores was considered as the reference group. Restricted cubic spline plots were used to evaluate trends among variables that were found to demonstrate the significance of logistic regression. They were also used to determine the presence of any non-linear association between exposure factors and OA risk. The potential mediating role of live microbes (in Lo, Med, and Hi foods) in the association between LE8 scores and OA risk was evaluated using a parallel mediation model. Mediation analysis was performed using the mediation package of R; a quasi-Bayesian Monte Carlo method with 1,000 simulations was used based on the normal approximation. The direct and indirect effects represented the effect of LE8 scores on OA risk without mediators and via the mediator, respectively. The proportion of mediation was calculated as the quotient of the indirect effect divided by the total effect.

All remaining statistical analyses were performed using R software (version 4.2.2, https://cran.r-project.org/bin/windows/base/old/4.2.2/); the following packages were used: nhanesR (version 0.9.2.8), survey, CompareGroups, dplyr, tidyverse, do, MASS, finalfit, Hmisc, lattice, Formula, rms, and foreign. The statistical tests were two-sided, and the results were considered statistically significant when the *P*-value was < 0.05.

## 3 Results

### 3.1 Baseline characteristics

The data from 23,213 participants (aged ≥20 years) were included. The baseline characteristics of the study population (according to OA risk) are presented in Table 1. The weighted percentages of participants aged 20–44, 45–64, and ≥65 years were 41.55%, 47.97%, and 37.72%, respectively. The weighted percentages of the 11,781 female and 11, 432 male individuals were 50.75% and 49.25%, respectively. The mean LE8 score for the total population was 68.40, and the low, moderate, and high scores (weighted %) were 5,022 (21.63), 1,688 (50.35), and 6,503 (28.01), respectively. The scores for those with OA (62.82) was lower than that of participants without OA (70.39). In contrast to those without OA, those with the condition were older, more likely to be women, and more likely to be of non-Hispanic white ethnicity; they also had lower LE8 (and its component metrics) scores, consumed more foods with lower live microbe content, and were mostly married. However, as shown in Table 1, live microbe intake (Med and Hi foods) and the ratio of family income to poverty levels did not differ significantly between the two groups.

**Table 1:** Participant demographic characteristics (NHANES, 2005–2019 years cycle).

**Parameter**	**No. of participants (weighted %)**			
	**Total**	**Non-osteoarthritis**	**Osteoarthritis**	***P-*value**
	**(*N* = 23,213)**	**(*N* = 16,709)**	**(*N* = 6,504)**	
CVH (Life's Essential 8)	68.40 (0.24)	70.39 (0.25)	62.82 (0.31)	< 0.001
Health behavior score	66.59 (0.32)	67.65 (0.31)	63.64 (0.49)	< 0.001
Health factor score	70.20 (0.24)	73.14 (0.26)	62.01 (0.30)	< 0.001
Score HEI	39.27 (0.49)	38.71 (0.51)	40.82 (0.68)	0.001
Score PA	71.96 (0.48)	75.16 (0.50)	63.01 (0.75)	< 0.001
Score smoke	71.55 (0.50)	72.24 (0.52)	69.61 (0.76)	< 0.001
Score sleep	83.59 (0.28)	84.48 (0.28)	81.11 (0.51)	< 0.001
Score BMI	60.53 (0.42)	63.59 (0.48)	51.98 (0.54)	< 0.001
Score non-HDL	64.30 (0.34)	65.95 (0.38)	59.69 (0.55)	< 0.001
Score glucose	86.29 (0.24)	89.06 (0.23)	78.55 (0.49)	< 0.001
Score BP	69.70 (0.35)	73.95 (0.39)	57.82 (0.56)	< 0.001
Grams Lo	3,447.93 (20.63)	3,486.76 (23.07)	3,339.56 (26.76)	< 0.001
Grams Med	108.52 (2.30)	108.04 (2.39)	109.86 (3.55)	0.590
Grams Hi	23.10 (0.66)	22.76 (0.67)	24.03 (1.30)	0.340
**Age, years old**				
20–44	9,645 (41.55)	8,831 (55.00)	814 (14.31)	< 0.001
45–64	8,103 (34.91)	5,314 (33.83)	2,789 (47.97)	
≥65	5,465 (23.54)	2,564 (11.18)	2,901 (37.72)	
**Sex**				
Female	11,781 (50.75)	7,962 (48.14)	3,819 (60.37)	< 0.001
Male	11,432 (49.25)	8,747 (51.86)	2,685 (39.63)	
**Ethnicity/ race**				
White people	10,915 (47.02)	7,239 (68.69)	3,676 (79.64)	< 0.001
Black people	4,663 (20.09)	3,327 (10.06)	1,336 (8.95)	
Mexican people	3,392 (14.61)	2,754 (8.80)	638 (3.66)	
Other	4,243 (18.28)	3,389 (12.44)	854 (7.74)	
**Marital**				
Married	14,114 (60.8)	10,343 (65.19)	3,771 (64.88)	< 0.001
Separated	5,040 (21.71)	2,852 (14.51)	2,188 (27.53)	
Never married	4,059 (17.49)	3,514 (20.29)	545 (7.59)	
**Ratio of family income to poverty levels**				
< 1.3	6,771 (29.17)	4,747 (18.74)	2,024 (19.68)	0.100
1.3–3	7,323 (31.55)	5,208 (27.86)	2,115 (29.71)	
3–5	4,688 (20.2)	3,491 (25.31)	1,197 (23.52)	
≥5	4,431 (19.09)	3,263 (28.10)	1,168 (27.09)	
**Education levels**				
Less than 11th grade	5,009 (21.58)	3,395 (12.86)	1,614 (16.12)	< 0.001
High school graduate	11,123 (47.92)	8,238 (55.75)	2,885 (50.47)	
College graduate or above	7,081 (30.5)	5,076 (31.40)	2,005 (33.41)	
**Alcohol consumption status**				
Never	3,836 (16.53)	2,296 (11.25)	1,540 (19.64)	< 0.001
Former	4,575 (19.71)	3,779 (23.28)	796 (13.08)	
Mild	8,080 (34.81)	5,665 (36.49)	2,415 (41.66)	
Moderate	3,703 (15.95)	2,813 (18.89)	890 (15.50)	
Heavy	3,019 (13.01)	2,156 (10.09)	863 (10.12)	
**CVH (Life's Essential 8)**				
Low	2,990 (12.88)	1,595 (7.69)	1,395 (17.55)	< 0.001
Moderate	15,696 (67.62)	11,170 (65.03)	4,526 (70.56)	
High	4,527 (19.5)	3,944 (27.28)	583 (11.89)	
**Health behavior score**				
Low	5,022 (21.63)	3,265 (17.31)	1,757 (23.32)	< 0.001
Moderate	11,688 (50.35)	8,452 (50.06)	3,236 (49.99)	
High	6,503 (28.01)	4,992 (32.63)	1,511 (26.69)	
**Health factor score**				
Low	4,024 (17.34)	2,175 (10.95)	1,849 (24.63)	< 0.001
Moderate	11,908 (51.3)	8,252 (48.00)	3,656 (56.67)	
High	7,281 (31.37)	6,282 (41.05)	999 (18.70)	

NHANES, National Health and Nutrition Examination Survey; FBG, fasting blood glucose; HEI score, Healthy Eating Index; Non-HDL, non-high-density lipoprotein cholesterol; BP, blood pressure; Experts assigned foods an estimated level of live microbes per gram [low (Lo), < 104 CFU/g; medium (Med), 104–107 CFU/g; or high (Hi), >107 CFU/g]; CVH, cardiovascular health.

Life's Essential 8, The components of Life's Essential 8 include diet (updated), physical activity, nicotine exposure (updated), sleep health (new), body mass index, blood lipids (updated), blood glucose (updated), and blood pressure. Each metric has a new scoring algorithm ranging from 0 to 100 points, allowing the generation of a new composite cardiovascular health score (the unweighted average of all components) that also varies from 0 to 100 points. The LE8 scoring algorithm consists of 4 health behaviors (diet, physical activity, nicotine exposure, and sleep duration) and 4 health factors [body mass index (BMI), non-high-density-lipoprotein cholesterol, blood glucose, and blood pressure]. The overall LE8 score and health factor score were calculated as the unweighted average of the eight metrics. Participants with a LE8 score of 80–100 were considered high CVH; 50–79, moderate CVH; and 0–49 points, low CVH.

Data are mean (standard error) or No. of participants (weighted %).

Percentages were adjusted for NHANES survey weights. The P-value was calculated using a chi-square test and Student's t-test after considering the sampling weights. P-value < 0.05 for each indicator, except for Grams Med, Grams Hi, Ratio of family income to poverty levels (P-value > 0.05).

HEI score, In the continuous US NHANES, healthy diet denoted the top two-fifths of the Healthy Eating Index-2015 score. Ethnicity/race: non-Hispanic white people, non-Hispanic Black people, Hispanic people, and others; Marital (married, separated, and never married).

### 3.2 Univariate logistic regression analysis for the association between LE8 scores and OA risk

Participants with low CVH (LE8 scores) demonstrated moderate [OR: 0.58; 95% CI: (0.51, 0.66)] and high [OR: 0.29; 95% CI: (0.25,0.34)] propensity for developing OA. Individuals with low health behavior scores also demonstrated moderate [OR: 0.75; 95% CI: (0.67, 0.84)] and high [OR: 0.55; 95% CI: (0.48, 0.62)] OA risk. Additionally, those with low health factor scores showed moderate [OR: 0.66; 95% CI: (0.58, 0.75)] and high [OR: 0.39; 95% CI: (0.34, 0.45)] likelihood of developing OA. The LE8, health behavior, and health factor scores remained significantly associated with OA risk on being used as continuous variables, and this was suggestive of their possible protective role against OA. Live microbe intake was associated with OA risk; in particular, the intake of Med [OR: 0.99; 95% CI: (0.99, 0.99)] and Hi [OR: 0.99; 95% CI: (0.99, 1.00)] category foods appeared to be protective against OA (Table 2).

**Table 2:** Univariate logistic regression analysis.

**Parameter**	**OR (95% CI)**	***P-*value**
CVH (Life's Essential 8)	0.97 (0.97, 0.97)	< 0.001
Health behavior score	0.99 (0.98, 0.99)	< 0.001
Health factor score	0.98 (0.98, 0.98)	< 0.001
Score HEI	0.99 (0.99, 0.99)	< 0.001
Score PA	0.99 (0.99, 0.99)	< 0.001
Score smoke	0.99 (0.99, 0.99)	< 0.001
Score sleep	0.99 (0.99, 0.99)	< 0.001
Score BMI	0.99 (0.99, 0.99)	< 0.001
Score non-HDL	0.99 (0.99, 0.99)	0.010
Score glucose	0.99 (0.99, 0.99)	< 0.001
Score BP	1.00 (1.00, 1.00)	0.090
Grams Lo	1.00 (1.00, 1.00)	0.690
Grams Med	0.99 (0.99, 0.99)	< 0.001
Grams Hi	0.99 (0.99, 1.00)	< 0.001
**CVH (Life's Essential 8)**		
Low	Ref	Ref
Moderate	0.58 (0.51, 0.66)	< 0.001
High	0.29 (0.25, 0.34)	< 0.001
**Health behavior score**		
Low	Ref	Ref
Moderate	0.75 (0.67, 0.84)	< 0.001
High	0.55 (0.48, 0.62)	< 0.001
**Health factor score**		
Low	Ref	Ref
Moderate	0.66 (0.58, 0.75)	< 0.001
High	0.39 (0.34, 0.45)	< 0.001

NHANES, National Health and Nutrition Examination Survey; FBG, fasting blood glucose; HEI score, Healthy Eating Index; Non-HDL, non-high-density lipoprotein cholesterol; BP, blood pressure; Experts assigned foods an estimated level of live microbes per gram [low (Lo), < 104 CFU/g; medium (Med), 104–107 CFU/g; or high (Hi), >107 CFU/g]; CVH, cardiovascular health; OR odds ratio, CI, confidence interval.

Life's Essential 8, The components of Life's Essential 8 include diet (updated), physical activity, nicotine exposure (updated), sleep health (new), body mass index, blood lipids (updated), blood glucose (updated), and blood pressure. Each metric has a new scoring algorithm ranging from 0 to 100 points, allowing the generation of a new composite cardiovascular health score (the unweighted average of all components) that also varies from 0 to 100 points. The LE8 scoring algorithm consists of 4 health behaviors (diet, physical activity, nicotine exposure, and sleep duration) and 4 health factors [body mass index (BMI), non-high-density-lipoprotein cholesterol, blood glucose, and blood pressure]. The overall LE8 score and health factor score were calculated as the unweighted average of the eight metrics. Participants with a LE8 score of 80–100 were considered high CVH; 50–79, moderate CVH; and 0–49 points, low CVH.

Adjusted for sex, age, ethnicity/race, marital, family income-to-poverty ratio, education levels, and alcohol consumption status.

### 3.3 Multivariate logistic regression analysis for the association between LE8 scores and OA

**Figure 2:**
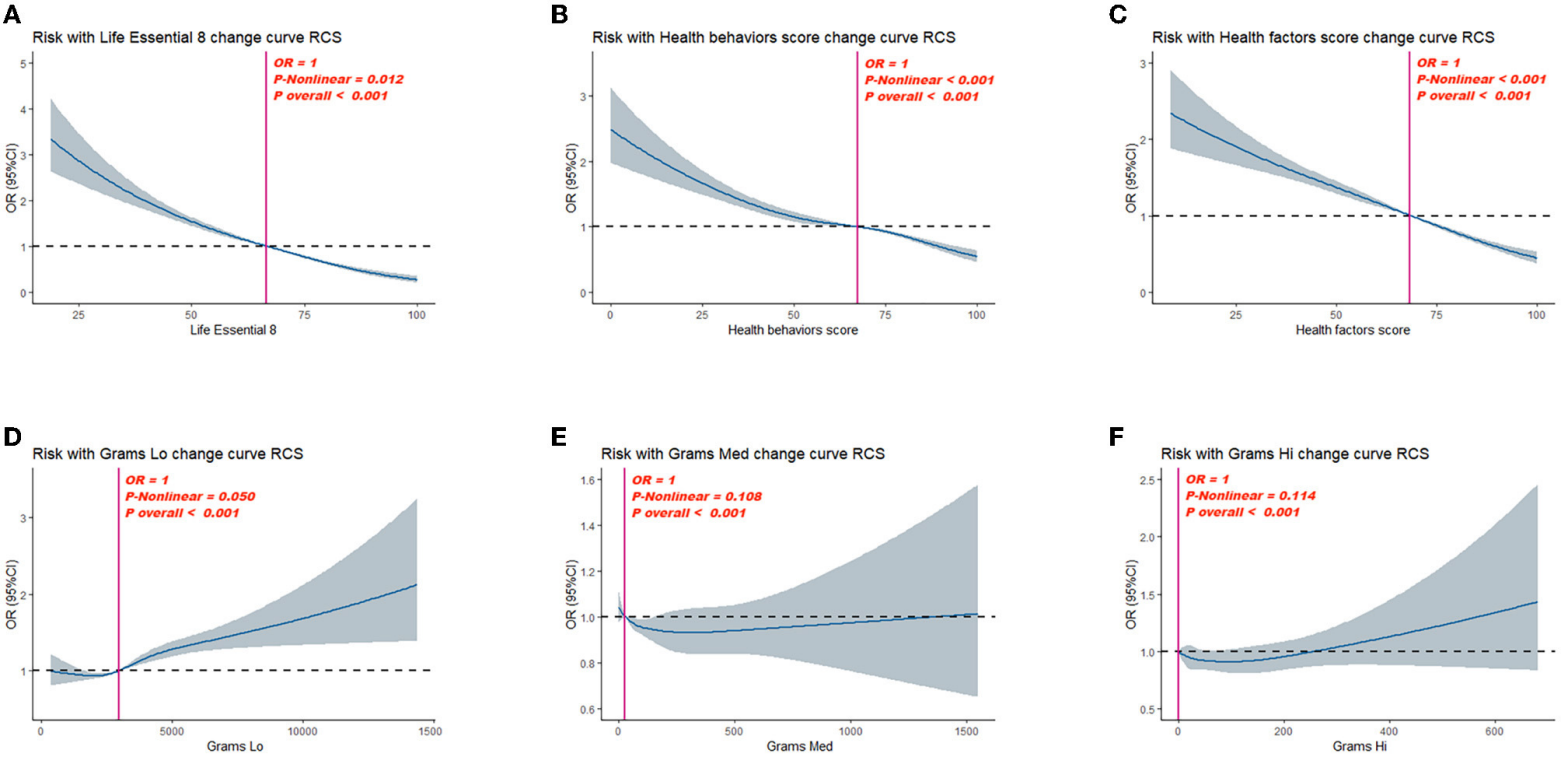
Dose–response relationships between Life's Essential 8 scores **(A)**, health behavior score **(B)**, health factor score **(C)**, Grams Lo **(D)**, Grams Med **(E)**, Grams Hi **(F)**, and Osteoarthritis (OA). OR (95% CI; shaded areas) were adjusted for sex, age, ethnicity/race, marital, family income-to-poverty ratio, education levels, and alcohol consumption status. Vertical red solid lines indicate the minimal threshold for the beneficial association with estimated OR = 1. OR, odds ratio.

After adjusting for multiple latent variables, LE8, health behavior, and health factor scores continued to demonstrate a significant association with OA risk. In model 2, the groups with moderate and high LE8 scores (representing CVH) demonstrated an OR of 0.81 (95% CI: 0.69, 0.96) for developing OA; in comparison, participants with low LE8 scores demonstrated an OR of 0.55 (95% CI: 0.44, 0.69). The groups with moderate and high health behavior scores demonstrated an OR of 0.83 (95% CI: 0.73, 0.95) for OA risk; in the group with low scores, the OR was 0.74 (95% CI: 0.63, 0.87). The groups with moderate and high health factor scores demonstrated an OR of 0.73 (95% CI: 0.64, 0.84), while the group with low scores showed an OR of 0.53 (95% CI: 0.45, 0.61; Table 3). As shown in Figure 2, a non-linear relationship was observed between OA risk and LE8 scores [*P*-nonlinear < 0.001; minimum threshold value for beneficial association: 66.52 (estimated OR = 1)], health behavior scores [*P-*nonlinear < 0.001; minimum threshold value for beneficial association: 67.34 (estimated OR = 1)], and health factor scores [*P*-nonlinear < 0.001; minimum threshold value for beneficial association: 68.36 (estimated OR = 1)].

**Table 3:** Multiple logistic regression models of Life's Essential 8 with osteoarthritis for participants.

**Parameter**	**Crude model**		**Model 1**		**Model 2**	
	**OR (95%CI)**	***P-*** **value**	**OR (95%CI)**	***P-*** **value**	**OR (95%CI)**	***P-*** **value**
**Life's Essential 8**						
Low	ref		ref		ref	
Moderate	0.75 (0.65, 0.87)	< 0.001	0.79 (0.67, 0.94)	0.010	0.81 (0.69, 0.96)	0.020
High	0.49 (0.40, 0.61)	< 0.001	0.53 (0.42, 0.66)	< 0.001	0.55 (0.44, 0.69)	< 0.001
*P*-trend	< 0.001					
**Health behavior score**						
Low	ref		ref		ref	
Moderate	0.87 (0.78, 0.97)	0.010	0.79 (0.70, 0.90)	< 0.001	0.83 (0.73, 0.95)	0.010
High	0.93 (0.81, 1.07)	0.310	0.69 (0.59, 0.80)	< 0.001	0.74 (0.63, 0.87)	< 0.001
*P*-trend	< 0.001					
**Health factor score**						
Low	ref		ref		ref	
Moderate	0.60 (0.53, 0.68)	< 0.001	0.73 (0.64, 0.84)	< 0.001	0.73 (0.64, 0.84)	< 0.001
High	0.28 (0.25, 0.32)	< 0.001	0.52 (0.44, 0.60)	< 0.001	0.53 (0.45, 0.61)	< 0.001
*P*-trend	< 0.001					

Life's Essential 8, The components of Life's Essential 8 include diet (updated), physical activity, nicotine exposure (updated), sleep health (new), body mass index, blood lipids (updated), blood glucose (updated), and blood pressure. Each metric has a new scoring algorithm ranging from 0 to 100 points, allowing the generation of a new composite cardiovascular health score (the unweighted average of all components) that also varies from 0 to 100 points. The LE8 scoring algorithm consists of 4 health behaviors (diet, physical activity, nicotine exposure, and sleep duration) and 4 health factors (body mass index [BMI], non-high-density-lipoprotein cholesterol, blood glucose, and blood pressure). The overall LE8 score and the health factor score were calculated as the unweighted average of the eight metrics. Participants with scores (LE8 score, health behavior score, and health factor score) of 80–100 were considered high CVH; 50–79, moderate CVH; and 0–49 points, low CVH.

OR odds ratio, CI confidence interval.

Crude model, No adjustment for any potential influence factors.

Model 1, Adjusted for sex, age, and ethnicity/race.

Model 2, Adjusted for sex, age, ethnicity/race, marital, family income-to-poverty ratio, education levels, and alcohol consumption status.

### 3.4 Subgroup analysis for the association between LE8 scores and OA risk

After adjusting for multiple latent variables, the LE8, health behavior, and health factor scores were all found to be significantly correlated with OA risk across the different groups (irrespective of sex, ethnicity/race, and age); therefore, these may serve as protective factors against OA. On comparing the group with low LE8 scores with the group having moderate and high scores, the ORs (with 95% CIs) for each subgroup were as follows: male: 0.55 (0.46, 0.66), 0.30 (0.23,0.38); female: 0.48 (0.40, 0.56), 0.16 (0.14,0.20); white: 0.54 (0.46, 0.64), 0.22 (0.18, 0.27); Black: 0.47 (0.40, 0.55), 0.17 (0.12, 0.24); Mexican: 0.21 (0.14, 0.32), 0.46 (0.34, 0.62); 20–44 years old: 0.37 (0.29, 0.48), 0.20 (0.14, 0.27); 45–64 years old: 0.37 (0.29, 0.48), 0.20 (0.14, 0.27), 0.20 (0.14, 0.27); 45–64 years old: 0.63 (0.53, 0.74), 0.25 (0.20, 0.31); and ≥65 years old: 0.75 (0.60, 0.93), 0.56 (0.41, 0.77). On comparing participants with low health behavior scores with those having moderate and high scores, the ORs (with 95% CIs) for each subgroup were as follows: male: 0.81 (0.69, 0.95), 0.70 (0.57, 0.86); female: 0.73 (0.63, 0.84), 0.55 (0.47, 0.64); white: 0.82 (0.71, 0.94), 0.63 (0.54, 0.74); Black: 0.74 (0.62, 0.90), 0.58 (0.45, 0.74); Mexican: 0.51 (0.40, 0.63), 0.49 (0.37, 0.65); 20–44 years old: 0.56 (0.46, 0.69), 0.41 (0.31, 0.55); 45–64 years old: 0.78 (0.67, 0.90), 0.51 (0.43, 0.61); and ≥65 years old: 1.01 (0.82, 1.23), 0.86 (0.69, 1.08). On comparing participants with low health factor scores with those having moderate and high scores, the ORs (with 95% CIs) for each subgroup were as follows: male: 0.60 (0.50, 0.71), 0.32 (0.26, 0.39); female: 0.52 (0.44, 0.62), 0.19 (0.16, 0.22); white: 0.57 (0.48, 0.68), 0.25 (0.22, 0.30); Black: 0.50 (0.43, 0.58), 0.19 (0.15, 0.24); Mexican: 0.49 (0.38, 0.64), 0.19 (0.14, 0.27); 20–44 years old: 0.46 (0.36, 0.59), 0.24 (0.19, 0.31); 45–64 years old: 0.69 (0.58, 0.81), 0.41 (0.35, 0.49); and ≥65 years old: 0.72 (0.60, 0.88), 0.57 (0.46, 0.72) (Table 4).

**Table 4:** Results of multiple logistic regression of participant scores using Life's Essential 8 with Osteoarthritis subgroup analysis.

**Parameter**	**Life's Essential 8**						**Health behavior score**						**Health Factor Score**					
	**Low**	**Moderate**		**High**		***P*** **-trend**	**Low**	**Moderate**		**High**		***P*** **-trend**	**Low**	**Moderate**		**High**		***P*** **-trend**
	**Ref**	**OR (95%CI)**	***P-*** **value**	**OR (95%CI)**	***P-*** **value**		**Ref**	**OR (95%CI)**	***P-*** **value**	**OR (95%CI)**	***P-*** **value**		**Ref**	**OR (95%CI)**	***P-*** **value**	**OR (95%CI)**	***P-*** **value**	
**Sex**																		
Male	Ref	0.55 (0.46, 0.66)	< 0.001	0.30 (0.23, 0.38)	< 0.001	< 0.001	Ref	0.81 (0.69, 0.95)	0.010	0.70 (0.57, 0.86)	< 0.001	< 0.001	Ref	0.60 (0.50, 0.71)	< 0.001	0.32 (0.26, 0.39)	< 0.001	< 0.001
Female	Ref	0.48 (0.40, 0.56)	< 0.001	0.16 (0.14, 0.20)	< 0.001	< 0.001	Ref	0.73 (0.63, 0.84)	< 0.001	0.55 (0.47, 0.64)	< 0.001	< 0.001	Ref	0.52 (0.44, 0.62)	< 0.001	0.19 (0.16, 0.22)	< 0.001	< 0.001
**Ethnicity/race**																		
White people	Ref	0.54 (0.46, 0.64)	< 0.001	0.22 (0.18, 0.27)	< 0.001	< 0.001	Ref	0.82 (0.71, 0.94)	0.004	0.63 (0.54, 0.74)	< 0.001	< 0.001	Ref	0.57 (0.48, 0.68)	< 0.001	0.25 (0.22, 0.30)	< 0.001	< 0.001
Black people	Ref	0.47 (0.40, 0.55)	< 0.001	0.17 (0.12, 0.24)	< 0.001	< 0.001	Ref	0.74 (0.62, 0.90)	0.002	0.58 (0.45, 0.74)	< 0.001	< 0.001	Ref	0.50 (0.43, 0.58)	< 0.001	0.19 (0.15, 0.24)	< 0.001	< 0.001
Mexican people	Ref	0.46 (0.34, 0.62)	< 0.001	0.21 (0.14, 0.32)	< 0.001	< 0.001	Ref	0.51 (0.40, 0.63)	< 0.001	0.49 (0.37, 0.65)	< 0.001	< 0.001	Ref	0.49 (0.38, 0.64)	< 0.001	0.19 (0.14, 0.27)	< 0.001	< 0.001
other	Ref	0.39 (0.29, 0.52)	< 0.001	0.15 (0.09, 0.26)	< 0.001	< 0.001	Ref	0.62 (0.46, 0.85)	0.004	0.51 (0.35, 0.73)	< 0.001	0.001	Ref	0.56 (0.41, 0.76)	< 0.001	0.16 (0.11, 0.25)	< 0.001	< 0.001
**Age, years**																		
20-44	Ref	0.37 (0.29, 0.48)	< 0.001	0.20 (0.14, 0.27)	< 0.001	< 0.001	Ref	0.56 (0.46, 0.69)	< 0.001	0.41 (0.31, 0.55)	< 0.001	< 0.001	Ref	0.46 (0.36, 0.59)	< 0.001	0.24 (0.19, 0.31)	< 0.001	< 0.001
45-64	Ref	0.63 (0.53, 0.74)	< 0.001	0.25 (0.20, 0.31)	< 0.001	< 0.001	Ref	0.78 (0.67, 0.90)	< 0.001	0.51 (0.43, 0.61)	< 0.001	< 0.001	Ref	0.69 (0.58, 0.81)	< 0.001	0.41 (0.35, 0.49)	< 0.001	< 0.001
≥65	Ref	0.75 (0.60, 0.93)	0.010	0.56 (0.41, 0.77)	< 0.001	< 0.001	Ref	1.01 (0.82, 1.23)	0.940	0.86 (0.69, 1.08)	0.200	0.130	Ref	0.72 (0.60, 0.88)	0.001	0.57 (0.46, 0.72)	< 0.001	< 0.001

OR, odds ratio; CI, confidence interval.

Life's Essential 8, The components of Life's Essential 8 include diet (updated), physical activity, nicotine exposure (updated), sleep health (new), body mass index, blood lipids (updated), blood glucose (updated), and blood pressure. Each metric has a new scoring algorithm ranging from 0 to 100 points, allowing the generation of a new composite cardiovascular health score (the unweighted average of all components) that also varies from 0 to 100 points. The LE8 scoring algorithm consists of 4 health behaviors (diet, physical activity, nicotine exposure, and sleep duration) and 4 health factors [body mass index (BMI), non-high-density-lipoprotein cholesterol, blood glucose, and blood pressure]. The overall LE8 score, health behavior score, and health factor score were calculated as the unweighted average of the 8 metrics. Participants with scores (LE8 score, health behavior score, and health factor score) of 80–100 were considered high CVH; 50–79, moderate CVH; and 0–49 points, low CVH.

Adjusted for sex, age, ethnicity/race, marital, family income-to-poverty ratio, education levels, and alcohol consumption status.

### 3.5 Mediation analysis

**Figure 3:**
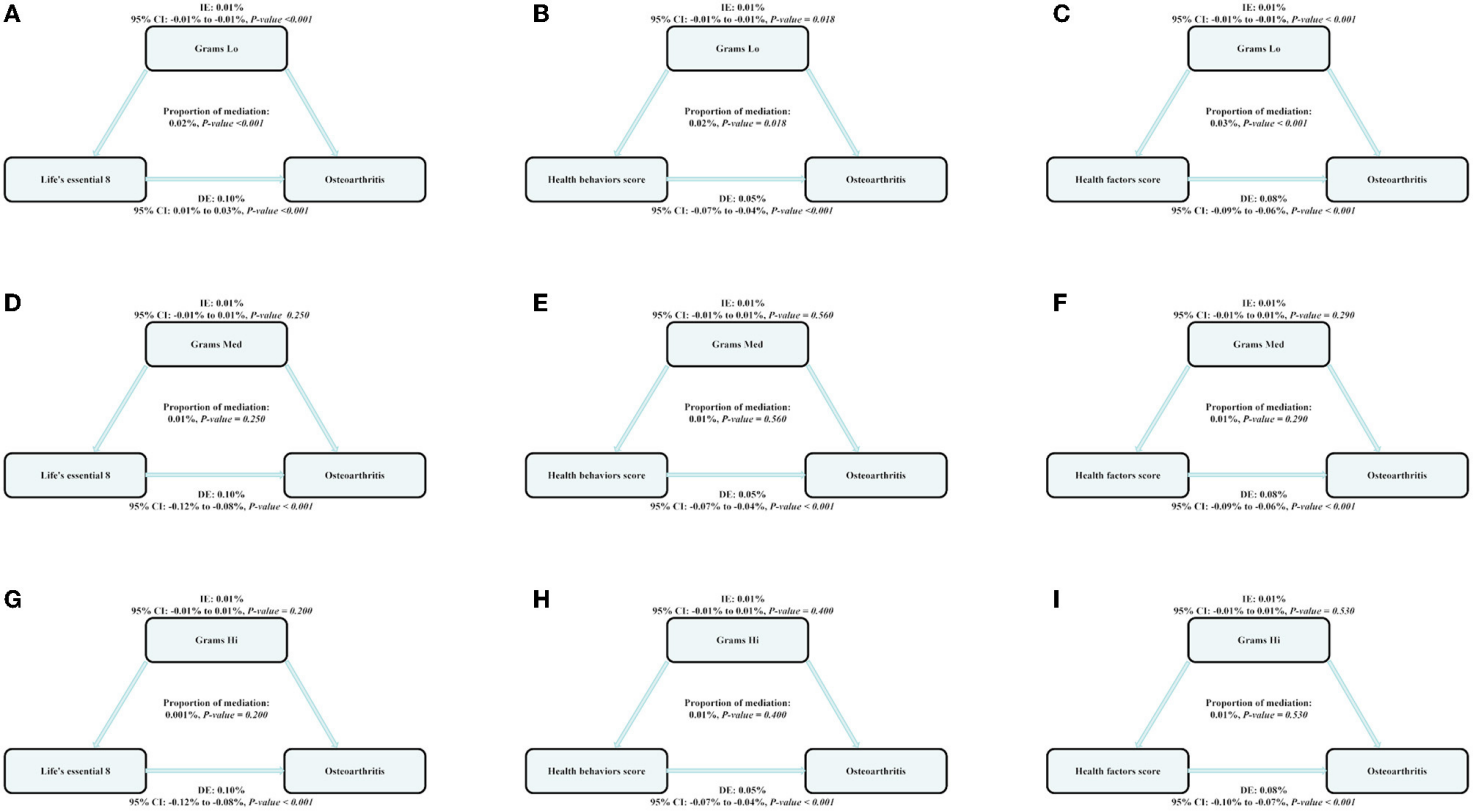
The estimated proportions of the associations between CVH (Life's Essential 8), health behavior score, health factor score, and OA mediated effect by the dose of dietary intake of live microbes. Model adjusted for sex, age, ethnicity/race, marital, family income-to-poverty ratio, education levels, and alcohol consumption status. IE, the estimate of the indirect effect; DE, the estimate of the direct effect; Proportion of mediation = IE/DE + IE, OR, odds ratio. **(A, D, G)** Shows the relationship between Life's Essential 8 and Osteoarthritis: the mediating effect of dietary live microbe (Grams Lo, Med, Hi) intake. **(B, E, H)** Shows the relationship between Life's Essential 8 and Osteoarthritis: the mediating effect of dietary live microbe (Grams Lo, Med, Hi) intake. **(C, F, I)** Shows the relationship between Life's Essential 8 and Osteoarthritis: the mediating effect of dietary live microbe (Grams Lo, Med, Hi) intake.

Parallel mediation analysis was performed to evaluate the potential mediating role of dietary active microorganisms in the association between the LE8, health behavior, and health factor scores and OA risk. Notably, Lo category foods demonstrated a mediating effect on the association between the LE8, health behavior, and health factor scores and OA risk. The mediation proportions were: 0.01%, *P* ≤ 0.001; 0.01%, *P* = 0.018; 0.01%; and *P* ≤ 0.001, respectively. A non-linear relationship was observed between Lo foods and OA risk (*P*-nonlinear = 0.050; Figure 2). As shown in Figure 3, the maximum threshold for the beneficial association was 2,972.81 CFU/g (estimated OR = 1).

## 4 Discussion

The present study provides two new major findings regarding the general population of the United States (based on the NHANES 2005–2019 cohort). First, LE8 scores consistently showed protective effects against OA. Second, Lo foods appeared to increase the risk of developing OA; in particular, they demonstrated a mediating effect in the protection offered by LE8 scores against OA.

Our findings suggest that higher LE8 scores reduce the risk of OA. In addition, the number of healthy LE8 indicators is directly proportional to the reduction in OA risk. In this context, direct research evidence on the association between LE8 scores and the risk of developing OA is currently lacking. Several studies have estimated the association between modifiable lifestyle-related risk factors and the risk of developing OA. Examples of such factors include smoking habits, dietary patterns, hypertension, sedentary lifestyle, BMI, low-density lipoprotein levels, exercise, and sleep (4–6, 21). CVH and OA are the most common causes of joint pain in adults with shared risk factors. As OA and CVD coexist, individuals who are at risk for developing one condition should be advised to undergo testing for the other (22). A study that evaluated four lifestyle factors (blood pressure, cholesterol levels, smoking, and diabetes) associated with CVD-related deaths before the age of 80 years, found that individuals with the best risk factor characteristics demonstrated a significantly lower risk than those with at least two major risk factors (23). Targeting these factors with low-intensity lifestyle interventions may therefore improve joint pain (24). Epidemiological studies have recognized healthy physical activity to be a key factor in the prevention and management of CVD and OA. A study also reported that 20–30 min of exercise performed once a week had a preventive effect on OA, especially in young patients with knee OA (25). Notably, insomnia and shorter sleep duration have an adverse impact on the risk of OA (21). Additionally, smoking, total counts, and triglyceride and low-density lipoprotein levels are risk factors for OA; an elevation in the levels of these laboratory parameters has been found to increase the risk of OA (26, 27). Emerging evidence suggests that hyperglycemia has a detrimental effect on the knee joint (28). The findings from studies have suggested that inflammation (29), lack of exercise (25), and medications (4) may contribute to this interrelationship. In this context, inflammatory mediators (including chemokines and cytokines) (30) play a key role in the pathogenesis of OA. In our study, higher LE8 scores (indicative of better CVH) consistently showed a protective effect against OA. Higher health behavior and factor scores were also found to be protective. This implies that a change in lifestyle habits may effectively reduce the risk of OA, and that CVH (as indicated by LE8 scores) has a non-negligible role in the prevention of OA.

Further mediation analyses were performed based on these findings. Foods in the Lo category demonstrated a significant mediating effect on the association between LE8 scores and OA risk; the mediation ratio was found to be significant at 0.01%. Based on our findings, the intake of Lo category foods with microbe numbers of < 2,972.81 CFU/g is likely to be associated with an increased risk of OA. Previous studies have reported that active probiotics can alleviate the symptoms of OA (31). In this context, interleukin-1β and tumor necrosis factor-α are secreted by synovial fibroblasts and chondrocytes in individuals with OA. These promote the synthesis of protein hydrolases, which degrade the joint extracellular matrix; this, in turn, drives disease progression, worsens disease-related synovial inflammation, and induces cartilage degeneration and the formation of subchondral bone lesions (32–34). Dietary probiotic supplementation promotes balance in the intestinal flora and reduces the inflammatory response, thereby reducing the risk of OA. Probiotics are effective in combating inflammation caused by interleukin-1β and tumor necrosis factor-α. Therefore, these cytokines represent important targets for probiotics (35–37) in the treatment of arthritis. The probiotics inhibit or reduce pro-inflammatory expression, thereby inhibiting joint degeneration (38, 39). In this context, *Clostridium butyricum* (GKB7 strain) has been found to produce butyrate, which can specifically increase mucin production and prevent microbes and their toxins from entering the circulation, and this reduces systemic inflammation (16). Probiotics may also reduce intestinal damage and inflammation associated with the OA disease process and have been found to reduce pain levels and cartilage destruction in animal models of OA (35, 40, 41). The GKB7 strain of *C. butyricum*, which is usually found in the environment, has also been found to ameliorate knee OA in rats (16). These findings suggest that dietary active microorganisms play an important role in preventing the development of OA. As foods in the Lo category contain fewer active microorganisms, diets incorporating a high proportion of Lo category foods may increase the risk of OA and weaken the protective effect of higher LE8 scores on OA.

The present study has several strengths. First, it assessed the relationship between LE8 scores and OA risk in a relatively large population. Second, it measured the dietary intake of live microbes and found macroscopic dietary live microbes to have a protective effect against OA and a mediating role in the association between LE8 scores and OA risk. This study also has certain limitations. First, self-reported OA diagnoses may reduce the validity of the results. Second, LE8 scores only represented important factors that influence the development of CVD; potential influences were not considered in this study. Third, estimates of live microbe intake were not measured for every sample, and this may have introduced bias. Fourth, residual and unmeasured confounding and measurement errors may have led to bias in our analysis. Fifth, although we adjusted for the survey period, the time span of our analysis was considerably long, and this may have led to bias. Sixth, we did not consider the influence of genetic factors. In this context, Zhang et al. proposed several central genes as possible biomarkers for OA diagnosis and these include the *POSTN, MMP 2, CTSG, ELANE, COL3A1, MPO, COL1A1*, and *COL1A2* (42) genes. Finally, we performed mediation analyses in a cross-sectional study, and this hindered the inference of causality. Given the limitations of the current study, the results need to be interpreted with caution. Further research is needed to support our findings.

## 5 Conclusion

In conclusion, LE8 scores play an important role in OA risk reduction. In our study, LE8 scores were found to be associated with live microbe intake, which reduced the risk of developing OA. Adherence to the LE8 recommendations and a reduction in Lo category food intake may be favorable for OA control. Our findings have helped identify protective factors against OA and the potentially detrimental effects of foods with low live microbe content.

## Data availability statement

Publicly available datasets were analyzed in this study. All data entered into the analysis were from NHANES, which is publicly accessible to all.

## Ethics statement

The surveys were approved by the NCHS Research Ethics Review Board (Protocol #2011-17). All methods were performed in accordance with the relevant guidelines and regulations (Declaration of Helsinki). Informed consent was obtained from all subjects and/or their legal guardian(s) (https://www.cdc.gov/nchs/nhanes/irba98.htm). The studies were conducted in accordance with the local legislation and institutional requirements. Written informed consent for participation was not required from the participants or the participants' legal guardians/next of kin in accordance with the national legislation and institutional requirements.

## Author contributions

RG: Conceptualization, Data curation, Formal analysis, Methodology, Software, Supervision, Writing—original draft, Writing—review & editing. XC: Supervision, Writing—review & editing. ZL: Supervision, Writing—review & editing. YP: Supervision, Writing—review & editing. GL: Funding acquisition, Supervision, Writing—review & editing.
